# The Mitochondrial Alternative Oxidase in *Ustilago maydis* Is Not Involved in Response to Oxidative Stress Induced by Paraquat

**DOI:** 10.3390/jof8111221

**Published:** 2022-11-19

**Authors:** Lucero Romero-Aguilar, Héctor Vázquez-Meza, Guadalupe Guerra-Sánchez, Oscar Ivan Luqueño-Bocardo, Juan Pablo Pardo

**Affiliations:** 1 Departamento de Bioquímica, Facultad de Medicina, Universidad Nacional Autónoma de México, Circuito Interior, Ciudad Universitaria, Coyoacán, Ciudad de México C.P. 04510, Mexico; 2 Instituto Politécnico Nacional, Escuela Nacional de Ciencias Biológicas, Departamento de Microbiología, Plan de Carpio y Plan de Ayala S/N Santo Tomás, Miguel Hidalgo, Ciudad de México C.P. 11340, Mexico

**Keywords:** alternative oxidase, glutathione, oxidative stress, paraquat, *Ustilago maydis*

## Abstract

It has been shown that the alternative oxidase in mitochondria of fungi and plants has important functions in the response against stress conditions, although their role in some organisms is still unknown. This is the case of *Ustilago maydis*. There is no evidence of the participation of the *U. maydis* Aox1 in stressful conditions such as desiccation, high or low temperature, and low pH, among others. Therefore, in this work, we studied the role of the *U. maydis* Aox1 in cells exposed to oxidative stress induced by methyl viologen (paraquat). To gain insights into the role of this enzyme, we took advantage of four strains: the FB2 wild-type, a strain without the alternative oxidase (FB2aox1Δ), other with the Aox1 fused to the Gfp under the control of the original promoter (FB2aox1-Gfp), and one expressing constitutively de Aox1-Gfp (FB2Potef:aox1-Gfp). Cells were incubated for various times in the presence of 1 mM paraquat and growth, replicative capacities, mitochondrial respiratory activity, Aox1 capacity, and the activities of several antioxidant enzymes (catalase, glutathione peroxidase, glutathione reductase, and superoxide dismutase) were assayed. The results show that (1) the response of *U. maydis* against oxidative stress was the same in the presence or absence of the Aox1; (2) the activities of the antioxidant enzymes remained constant despite the oxidative stress; and (3) there was a decrease in the GSH/GSSG ratio in *U. maydis* cells incubated with paraquat.

## 1. Introduction

*Ustilago maydis* is a dimorphic fungus that infects maize plants and induces the formation of galls and tumors [1]. In the last two decades, *U. maydis* has become a significant eukaryotic model for studying cell processes such as pathogenicity [1,2,3], signal transduction pathways involved in the dimorphic transitions and plant infection [3], cell physiology [4,5,6,7], intermediary metabolism [8], and the heterologous production of proteins and lipids [9,10]. *U. maydis* is an aerobic microorganism with the classic mitochondrial cytochrome pathway, a cyanide-resistant alternative oxidase (Aox1), and three alternative dehydrogenases [4,6,11]. The AOX branches the electron flow at the ubiquinone level by coupling the direct oxidation of ubiquinol with the reduction of O_2_ to H_2_O. In this way, this enzyme allows for the activity of the Krebs cycle when the cytochrome pathway is blocked or stimulates heat dissipation in the flowers of thermogenic plants to attract insects for pollination [12]. AOX activity is stimulated by low-energy conditions such as the inhibition of the respiratory chain by cyanide or antimycin A, or environmental low-oxygen concentrations, both stressful conditions in which there is an increase in reactive oxygen species (ROS) [5]. A diagnostic property of fungal and plant AOXs is their resistance to the classic inhibitors of the respiratory chain such as rotenone, cyanide, nitric oxide, azide, and antimycin A, and the inhibition by *n*-octyl gallate (nOG) and SHAM [13,14]. 

One of the main functions of AOX in plants and fungi is to decrease the production of ROS and counteract several types of stress, but mostly oxidative stress [15,16,17]. Therefore, the suggestion that high AOX activities are associated with the defense of organisms against environmental and physiological stresses is reasonable. However, in the phytopathogen *U. maydis*, the Aox1 did not take part in the response of the cell against high or low temperatures or desiccation [7], suggesting that the role of this enzyme in cell survival under these extreme conditions is minor. Furthermore, the lack of Aox1 did not affect the growth of *U. maydis*, since both vegetative and filamentous growth was the same in the wild-type and FB2aox1Δ [7]. Surprisingly, and given the important roles of the AOX in the infection process in many pathogenic microorganisms [14], the infectivity of *U. maydis* was not decreased in FB2aox1Δ [7]. However, this behavior has been observed in other phytopathogens that belong to the Ascomycota or Basidiomycota division such as *Magnaporthe grisea*, a Sordariomycete that infects rice [18,19], and *Sporisorium reilianum f. sp*. *zeae*, a Microbotryomycete able to infect maize [20]. Like *U. maydis*, the ability of these fungi to infect the respective plants was not affected by the lack of AOX. 

The goal of this work was to gain insights into the role of the Aox1 in *U. maydis* cells subjected to oxidative stress. To achieve this objective, four strains with different degrees of Aox1 expression and promoter regulation were used: FB2 wild-type, FB2aox1Δ, FB2aox1-Gfp, and FB2Potef:aox1-Gfp. The last mutant constitutively expressed Aox1 and according to some studies in the literature, it should be more resistant to oxidative stress. To induce oxidative stress in cells, we used methyl viologen (paraquat), a herbicide commonly used in farm fields. Paraquat is an efficient producer of ROS inside the cells [21,22]. In the first step, the divalent cation paraquat (PQ^2+^) accepts an electron in reactions catalyzed by oxidoreductases containing FAD or FMN as prosthetic groups such as NADPH oxidases or complex I in the inner mitochondrial membrane, and then, the paraquat radical (PQ^+^) reacts with molecular oxygen (O_2_) to form the superoxide anion (O_2_^−^), which is the precursor of hydrogen peroxide (H_2_O_2_) and other ROS [23,24,25]. Because the responses of organisms to stress imply the expression and repression of many genes [26,27], we also assayed the activity of some of the enzymes involved in the detoxification system such as superoxide dismutase (SOD), catalase (CAT), and the glutathione peroxidase (GPx) and reductase (GR) [28]. 

## 2. Materials and Methods

### 2.1. Reagents

Methyl viologen (1,1′-dimethyl-4-4′-bipyridinium dichloride, paraquat) (referred here as PQ), Calcofluor-White M2R, reduced and oxidized glutathione, glutathione reductase, cumene hydroperoxide, and 2,7-dichlorofluorescein diacetate (DCFH-DA) were purchased from Sigma-Aldrich. Mitotracker Green FM was purchased from Thermo Fisher. Other reagents used in this work were of analytical grade and obtained from Sigma-Aldrich. A stock solution of 720 mM of PQ was prepared in sterile distilled water and stored at 4 °C. Mitotracker Green FM was prepared as 1 mM stock solution in DMSO and stored at −20 °C. DCFH-DA was prepared as a 10 mM solution in DMSO and stored at −20 °C. Calcofluor-White M2R was prepared immediately before use at 100 ng/mL with sterile distilled water. 

### 2.2. Strains and Growth Conditions

*Ustilago maydis* wild-type FB2 (a2b2), FB2aox1-Gfp, FB2Potef:aox1-Gfp, and FB2aox1Δ were maintained in 25% glycerol (*v/v*) at −70 °C and recovered in Yeast Peptone Dextrose Agar (YPD-agar) (0.5% yeast extract, 0.25% bactopeptone, 0.5% glucose, and 2% agar). Cells were cultured in 50 mL of YPD (0.5% yeast extract, 0.25% bactopeptone, 0.5% glucose) medium for 24 h at 180 rpm and 28 °C, recovered by centrifugation for 5 min at 3000× *g* at 4 °C, and suspended in sterile distilled water (1 mL H_2_O per g wet weight). These cells (pre-cultured cells) were used as the inoculum for subsequent experiments.

### 2.3. Cell Viability

Pre-cultured cells were inoculated at an optical density (OD_600 nm_) of 0.5 in the YPD medium with or without 1 mM of PQ and incubated for 40 min at 28 °C and 180 rpm. Cells were harvested and resuspended with sterile distilled water to an OD_600 nm_ of 0.5. Then, serial dilutions were carried out and aliquots of 2 μL were dropped on solid YPD-agar. Plates were incubated at 28 °C and observed at 24, 32, and 48 h. 

### 2.4. Oxygen Consumption 

Fifty OD_600 nm_ units of pre-cultured cells were inoculated into 10 mL of YPD (final OD_600 nm_ = 5) with or without 1 mM PQ and incubated at 28 °C and 180 rpm. Aliquots of 2 mL were withdrawn at the indicated times (1, 2, 3, 4 h), centrifuged at 3000× *g* and 4 °C. Samples were resuspended to a final volume of 130 µL. Aliquots of 20 µL were used to assay the respiratory activity of cells. Oxygen consumption was measured with a Clark-type electrode in 1.2 mL of a buffer containing 20 mM 4-(2-hydroxyethyl)-1-piperazineethanesulfonic acid (HEPES) adjusted to pH 7.0 with 2-amino-2-(hydroxymethyl)-1,3-propanediol (Tris). To inhibit the activity of mitochondrial respiratory enzymes, 1.5 mM potassium cyanide (KCN) (complex IV inhibitor) and 6.0 µM *n*-octyl gallate (nOG) (AOX inhibitor) were used. The oxygen consumption rate is reported as nmol O_2_ (min·mg dry weight)^−1^. 

### 2.5. Flow Cytometry

Cells FB2aox1-Gfp was incubated for 4 h at 28 °C and 180 rpm in the presence or absence of 1 mM PQ, as described under the “Oxygen consumption” Section 2.4. Then, the control and PQ-treated cells were fixed with 4% formaldehyde (15 min at room temperature) and washed twice with 0.9% sodium chloride (NaCl) solution. For acquisition, cells were resuspended to a final concentration of 5 × 10^6^ cells/mL, vortexed heavily, and briefly sonicated in a compact high performance ultrasonic cleaning system (Fisher Scientific FS3). Per condition, 20,000 events were acquired by the flow cytometer MACSQUANT analyzer 10 (Miltenyi Biotec, Bergisch Gladbach, Germany). Four samples from independent cultures were analyzed. 

### 2.6. Production of Hydrogen Peroxide by U. maydis Cells

Production of hydrogen peroxide (H_2_O_2_) by *U. maydis* cells was measured with the Amplex™ Red Kit from Thermo Fisher Scientific. Ten mL of fresh YPD media with or without 1 mM PQ was inoculated with 50 U of pre-cultured cells (final OD_600 nm_ of 5) and incubated for 4 h at room temperature. At the end of the incubation, 50 µL of the cell suspension was mixed with 50 µL of the Amplex™ Red Kit solution, prepared according to the supplier’s specifications. Production of H_2_O_2_ was followed using a Thermo Scientific™ Varioskan™ Lux multimode microplate reader (excitation at 530 nm and emission at 590 nm) for 1.7 h with cycles of 30 s at room temperature and shaking between each cycle. 

### 2.7. Detection of Intracellular Reactive Oxygen Species

Detection of H_2_O_2_ was performed according to Kretschmer M et al. (2018) [29] with minor modifications. *U. maydis* strains were cultured in YPD for 24 h at 28 °C. Cells were collected by centrifugation for 5 min at 3000× *g* at 4 °C and resuspended with sterile distilled water. Five mL of YPD or YPD plus 1 mM PQ were inoculated with 4.6 × 10^6^ cells/mL (0.2 UA) and incubated for 2 h at 28 °C and 200 rpm. The cells were collected by centrifugation, as described above, resuspended at 5 × 10^6^ cells/mL with minimal medium containing 100 µM of DCFH-DA, and incubated for 1 h at 37 °C in the darkness with continuous shaking [29]. At the end of the incubation, cells were collected and washed once with a minimal medium. Confocal microscopy was performed as described below.

### 2.8. Confocal Microscopy

The *U. maydis* strain with the Aox1-Gfp construction and stained with DCFH-DA was used for the confocal microscopy experiments. Cells incubated for 4 h in the presence or absence of 1 mM PQ were harvested, washed once with sterile distilled water, and resuspended to an OD_600 nm_ of 5. Then, cells were mounted on Silane-Prep Slides (Sigma-Aldrich, St. Louis, USA), and imaged on a confocal microscope (Zeiss LSM5 Pascal, Carl Zeiss GmbH, Göttingen, Germany) with an oil-immersion 100 X N.A. 1.3 objective. Images were analyzed with Fiji software [30]. 

### 2.9. Preparation of Cell-Free Extracts

Cells incubated for 4 h in the presence or absence of 1 mM PQ were harvested and resuspended with the following lysis buffer: 50 mM monopotassium phosphate (KH_2_PO_4_), 30 mM HEPES, 5 mM ethylenediaminetetraacetic acid (EDTA), 20% glycerol, 1 mM phenylmethylsulfonyl fluoride (PMSF), pH 7.0, and mixed with one volume of glass beads (0.5 mm). Cells were subjected to five pulses of 1 min at 4800 oscillations/min using the mini bead-beater equipment with intervals of 1 min incubation in an ice bath. The lysate was clarified by centrifugation for 20 min at 20,000× *g* at 4 °C in a SCILOGEX SCI24R high-speed refrigerated microcentrifuge. 

### 2.10. Glutathione Reductase 

The activity of the glutathione reductase was determined by following the oxidation of nicotinamide adenine dinucleotide phosphate reduced (NADPH) at 340 nm. The assay contained 20 mM phosphate buffer (NaH_2_PO_4_/Na_2_HPO_4_), 1 mM EDTA, 0.15 mM NADPH, 1 mM oxidized glutathione (GSSG), pH 7.0. The reaction was started by adding the cell extract. The specific activity was calculated using an extinction coefficient of 6.22 mM^−1^ cm^−1^ for the NADPH and considering the protein in the assay [31]. 

### 2.11. Glutathione Peroxidase 

Glutathione peroxidase activity was assayed by following the oxidation of the NADPH at 340 nm. The assay had 20 mM phosphate buffer (NaH_2_PO_4_/Na_2_HPO_4_), 1 mM EDTA, 0.15 mM NADPH, 1 mM reduced glutathione (GSH), 0.1 mM cumene hydroperoxide, and pH 7.0. Adding the cell extract started the reaction. The specific activity was calculated using an extinction coefficient of 6.22 mM^−1^ cm^−1^ for the NADPH and the protein in the assay [32].

### 2.12. Superoxide Dismutase 

Superoxide dismutase (SOD) was determined as the percent inhibition of nitroblue tetrazolium (NBT) reduction. NBT reduction was followed at 560 nm. The buffer assay contained 0.2 mM NBT, 0.1 mM EDTA, 0.6% Triton^TM^-X 100, 50 mM sodium carbonate (Na_2_CO_3_), and 20 mM hydroxylamine hydrochloride (NH_2_OH.HCl). The final pH of 7.0 [33] was incubated for ten minutes at 37 °C. Then, an aliquot of the cell extract (20 μL) was added, and readings were recorded for 7 min. 

### 2.13. Catalase-Peroxidase 

The catalase-peroxidase activity was determined by following the disappearance of H_2_O_2_ at 240 nm. The reaction mixture contained 20 mM phosphate buffer (NaH_2_PO_4_/Na_2_HPO_4_), pH 7.0, and 30 mM H_2_O_2_. The reaction was started with the addition of an aliquot of the cell extract (10 μL). 

### 2.14. Glutathione and Glutathione Disulfide Determination

Glutathione was determined according to Rashman I et al. (2006) and Akerboom TP et al. (1981) [34,35]. *U. maydis* cells were treated with or without 1 mM PQ for 4 h and collected by centrifugation at 10,000 rpm for 1 min at 4 °C in a toptable centrifuge. Four volumes of 0.72 M perchloric acid (HClO_4_) were added to the sample (4 mL/g wet weight), and vortexed vigorously. After 5 min on ice, the sample was centrifuged at 5000 rpm for 5 min at 4 °C. The supernatant was recovered, and as quickly as possible, the pH was adjusted to a value between 4.5 and 6 using 2 M potassium hydroxide (KOH) and centrifuged at 5000 rpm for 5 min at 4 °C. For the determination of glutathione disulfide (GSSG), 6 μL of a 10-fold dilution of 2-vinylpyridine was added to 200 μL of the supernatant and incubated for 2 h in the dark at room temperature. At acidic pH, oxidation of GSH is inhibited and the reaction of 2-vinylpyridine with GSH allows for the determination of GSSG. Glutathione disulfide and the total glutathione were determined enzymatically by following the production of 2-nitro-5-mercapto-benzoic acid at 412 nm. The reaction mixture contained 100 mM phosphate buffer pH 7.0, 1.4 U of glutathione reductase, 1 mM EDTA, and 80 μM DTNB. A 10 μM GSSG solution was used for the standard curve. The GSH was obtained by subtracting the GSSG from the total glutathione. 

### 2.15. Statistical Analysis

Statistical analysis of data were performed in GraphPad Prism V.6b (Trial). A one-way ANOVA test was used to analyze the data. A statistical significant difference was defined for a *p* ≤ 0.05.

## 3. Results

### 3.1. Effect of Paraquat on U. maydis Growth

**Figure 1:**
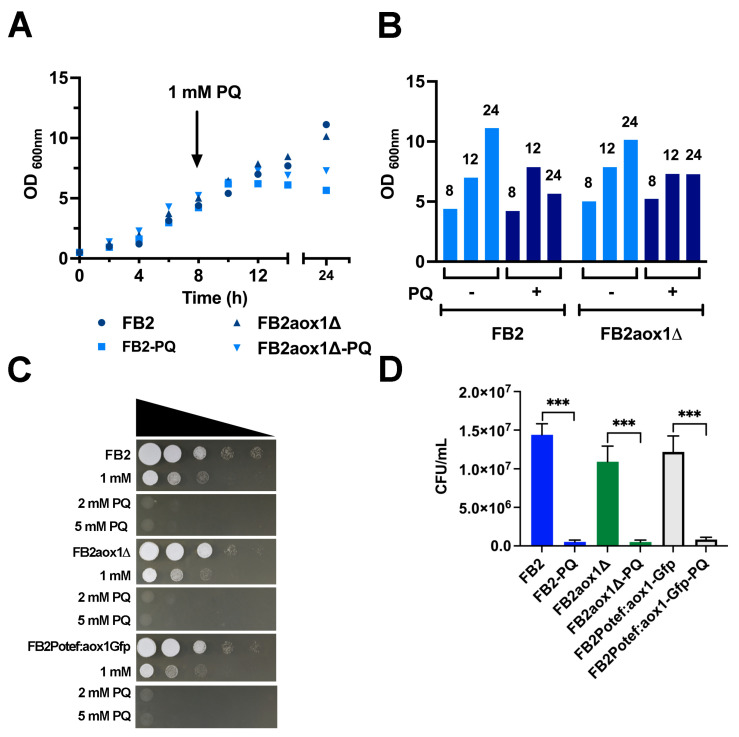
Inhibition of *U. maydis* growth by PQ. (**A**). Cells were inoculated in fresh YPD media at an initial concentration of 0.05 OD_600 nm_ and incubated at 28 °C and 200 rpm. At the 8 h point, 1 mM PQ (final concentration) was added to the culture media, and cell growth was followed by measuring the OD_600 nm_. The data are the mean of two experiments. (**B**) For clarity, the data points of 8, 12 and 24 h were displayed in a bar plot. (**C**) The spot assay was carried out with cells incubated for 4 h in the presence or absence of different concentrations of PQ (0, 1, 2, and 5 mM), harvested, and resuspended at 0.5 OD_600 nm_. The 10-fold serial dilutions were prepared and spotted on YPD agar. The figure shows a representative result of four independent experiments (*n* = 4). (**D**) The colony forming units per mL (CFU/mL) were estimated in cells incubated in the presence or absence of 1 mM PQ. Vertical bars indicate the standard error from at least four independent experiments (*n* = 4–5). *** Indicates a significant difference at *p* < 0.05.

Because PQ is highly toxic to eukaryotic and prokaryotic cells [22,36,37,38,39], the tolerance to PQ of four *U. maydis* strains with different contents of Aox1 was analyzed: the wild-type FB2, a strain without the *aox1* gene (FB2aox1Δ), a strain expressing a C-terminal Gfp fusion of Aox1 under the control of the native *aox1* promoter (FB2aox1-Gfp), and a strain expressing the Aox1-Gfp under the constitutive Potef promoter (FB2Potef:aox1-Gfp). First, FB2 and FB2aox*1*Δ strains were grown in YPD media, and PQ was added at 8 h; the growth was followed for 16 additional hours. As shown in Figure 1A, there was an increase in OD_600 nm_ in the next 4 h after the addition of PQ (8 to 12 h), indicating the ability of the cells to grow under these conditions. However, after 4 h in the presence of PQ, cells stopped growing. This can be seen more clearly in Figure 1B. From 8 to 12 h, there was an increase in OD_600 nm_ regardless of the presence of PQ, but from 13 to 24 h, the increase in OD stopped when cells were in contact with PQ (Figure 1B).

Next, we studied the effect of PQ on the cell growth and replicative capacity of cells. After incubation of the strains with different concentrations of PQ [0 mM, 1 mM, 2 mM, and 5 mM], 2 μL aliquots from 10-fold serial dilutions were dropped on solid YPD-agar and incubated for 24 h at 28 °C. The growth of the three strains (FB2, FB2aox1Δ, and FB2Potef:aox1-Gfp) was negatively affected by the PQ treatment in the same proportion (Figure 1C). Similar decrements in growth were seen at 1 mM even though the Aox1 was constitutively expressed or absent. The higher the concentration of PQ, the larger the inhibition of growth (Figure 1C). When the replicative capacity of cells was assayed by counting the colonies produced by *U. maydis* cells after incubation for 1.5 h in the presence of PQ, we found that more than 95% of the cells lost their capacity to duplicate (Figure 1D). Taken together, the results suggest that the damage induced by PQ to cell structures such as the chemical modification of nucleotides in DNA and fatty acids in membranes [40,41] is sufficient to stop cellular replication, and this damage is independent of the presence of Aox1.

### 3.2. Paraquat and the Mitochondrial Respiratory Activity

**Figure 2:**
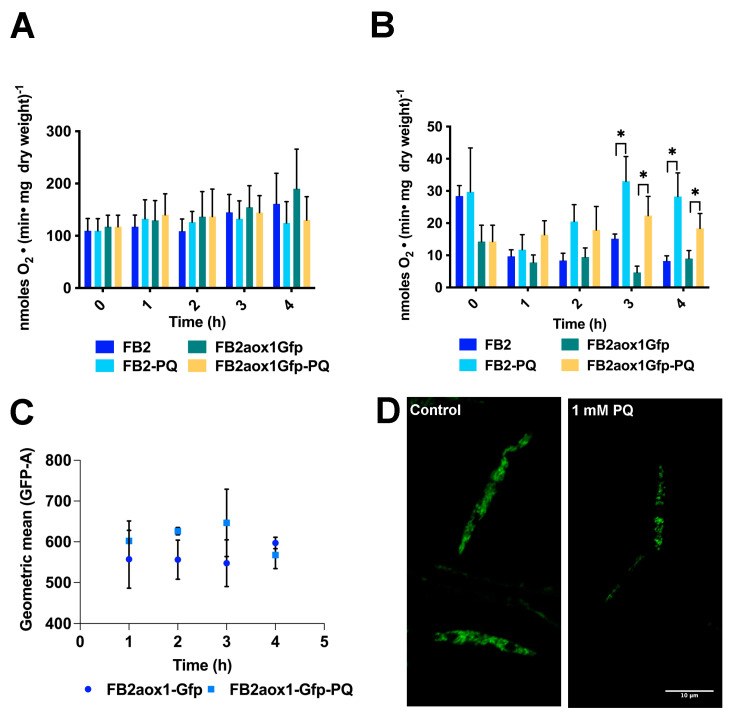
Effect of PQ on the activity and amount of *U. maydis* Aox1. A cellular suspension with an initial OD_600 nm_ of 5 was incubated for 4 h in the presence or absence of 1 mM PQ. At the indicated times, cells were harvested and resuspended in water (1 mL H_2_O:1 g wet weight). Oxygen consumption was analyzed with a Clark-type electrode in the absence (**A**) or presence (**B**) of 1.5 mM cyanide, an inhibitor of the cytochrome pathway. (**C**) Changes in Aox1 expression were determined by measuring the fluorescence of the Aox1-Gfp. Samples were withdrawn at the indicated times, and the fluorescence was determined by flow cytometry in 20,000 events per condition. (**D**) The changes in fluorescence were corroborated by confocal microscopy. A one-way ANOVA was performed to detect significant differences among the means. * Significant difference at *p* < 0.05. The data correspond to an average of four independent experiments (*n* = 4) with bars indicating the standard error.

The one-electron reduction of PQ by complex I in the respiratory chain is associated with the production of superoxide anion (O_2_^−^) and H_2_O_2_ in the mitochondrial matrix [24,39], raising the possibility of the inhibition of the respiratory complexes. Therefore, we studied the respiratory activity of cells incubated in the presence or absence of 1 mM of PQ, taking aliquots at the indicated times to measure oxygen consumption. In contrast to the expectation, there was not a time-dependent decrease in the respiratory activity of cells incubated in the presence of PQ (Figure 2A,B). Additionally, the respiratory activity of cells in the presence or absence of PQ was the same, within the experimental error. The results suggest that the production of ROS by PQ in the *U. maydis* mitochondrial matrix did not affect the functioning of the respiratory complexes (Figure 2A,B).

### 3.3. Paraquat and the Aox1 Activity

Reports in the literature have shown that the incubation of cells with PQ or H_2_O_2_ causes an increase in cyanide-resistant respiration in fungal and plant cells [36]. To further study the participation of Aox1 in the response of *U. maydis* to oxidative stress, the cyanide-resistant respiratory capacity of cells was determined. To measure the respiratory activity of the cells, two strains were selected, FB2aox1-Gfp and FB2. Aox1 capacity was obtained by adding 1 mM cyanide and then 6 μM nOG. The residual respiratory activity resistant to cyanide and inhibited by nOG defines the oxygen consumption given by the Aox1. The results showed that in the first hour of incubation, there was a decrease in the activity of Aox1, independent of the presence of PQ. This decrease in activity when the cells are transferred to fresh YPD medium has previously been reported [7]. From this point, PQ induced a time-dependent increase in cyanide-resistant respiration. When compared with the control condition, the increase in Aox1 activity was the most notorious and statistically significant at the 3 and 4 h points (Figure 2A,B). The larger Aox1 activity in the presence of PQ can be explained either by activation of Aox1 at the protein level or by activation of gene expression. To evaluate the second possibility, we took advantage of the fluorescence emitted by the FB2aox1-Gfp strain and carried out the quantification of the protein by flow cytometry (Figure 2C). The results show that the amount of Aox1-Gfp in the control cells and PQ-treated cells were similar within the experimental error (Figure 2C). The difference in the mean fluorescence intensities of GFP (GEO-MFI-GFP) between the control (597 ± 18) and the PQ-treated cells (568 ± 19) was not significant. The similarity in the GEO-MFI-GFP values was corroborated by confocal microscopy (Figure 2D). The micrographs in Figure 2D show that the fluorescence was approximately the same in cells incubated in the absence or presence of PQ, suggesting that under oxidative stress, there was no increase in the expression of Aox1. However, a closer look at the cells incubated with PQ shows that the fluorescence is punctuated, suggesting mitochondrial fragmentation (Figure 2D). This result is in agreement with several reports describing the effect of H_2_O_2_ on mitochondrial structure [42]. The results indicate that treatment with PQ induced a small increase in Aox1 activity, which was not due to the higher Aox1 expression. In addition, PQ induced fragmentation of mitochondria, probably because of the increased production of H_2_O_2_.

### 3.4. Production of H_2_O_2_ in Cells Treated with Paraquat

**Figure 3:**
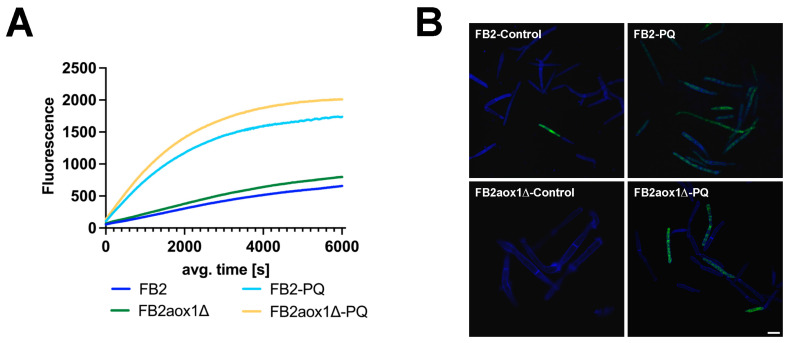
Production of H_2_O_2_. Release of H_2_O_2_ into the culture medium was quantified with the Amplex Red Kit (**A**). The increase in mitochondrial H_2_O_2_ production by cells incubated in the presence of 1 mM PQ was observed by confocal microscopy (**B**). H_2_O_2_ production by mitochondria was detected with DCFH-DA.

It is known that PQ generates oxidative stress in many types of cells [21,22,36]. The transfer of one electron from the PQ radical (PQ^+^) to O_2_ generates the O_2_^−^, which in turn dismutates to H_2_O_2_ [25]. Therefore, the next step was to fluorometrically measure the production of H_2_O_2_ inside the *U. maydis* cells and the release of this species into the extracellular medium. H_2_O_2_ released by *U. maydis* cells was measured with the Amplex^TM^ Red Hydrogen Peroxide Assay Kit. Figure 3A shows that in the absence of PQ, both strains, FB2 and FB2aox1Δ, released H_2_O_2_ into the extracellular space. However, in the presence of PQ, there was a 10-fold increase in the initial rate of H_2_O_2_ production, from 55 RFU (relative fluorescence units) to 660 RFU for FB2, and 68 RFU to 790 RFU for FB2aox1Δ (Figure 3A). It is worth noting that the efflux of H_2_O_2_ was similar in both strains, suggesting that the production of H_2_O_2_ inside the cell was similar regardless of the presence of Aox1. Next, we asked whether the release of H_2_O_2_ was associated with an increase in the intracellular production of H_2_O_2_. H_2_O_2_ in *U. maydis* cytosol was detected by confocal microscopy using the fluorescent properties of DCFH-DA. Figure 3B shows that the fluorescence inside the cells increased with the incubation with PQ (Figure 3B), and the response was similar for the FB2 and FB2aox1Δ strains. The results show that PQ induces an increase in the production of H_2_O_2_ inside the cell, which in turn is released into the extracellular medium.

### 3.5. Effect of Paraquat on the Activity of Antioxidant Enzymes

**Figure 4:**
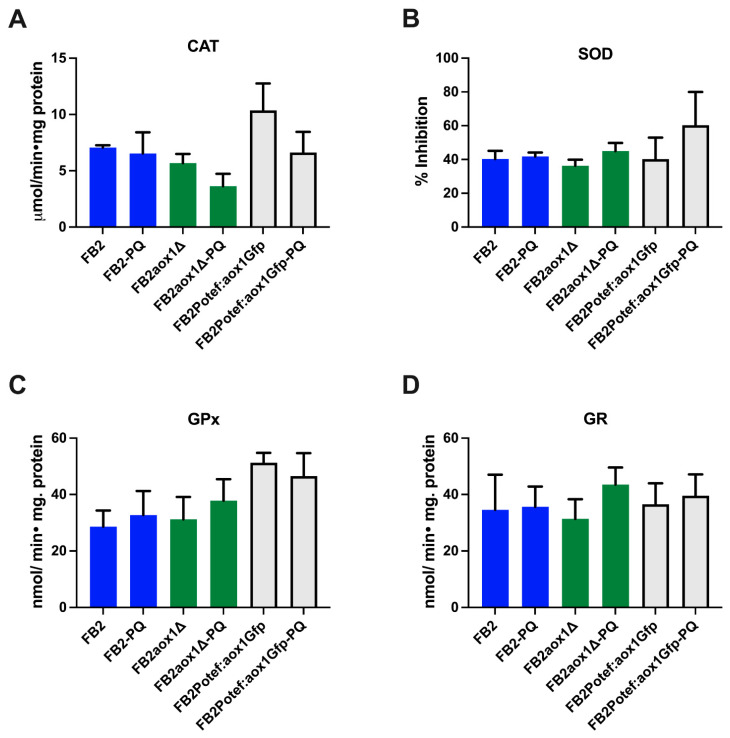
Activity of the antioxidant enzymes. The activity of: (**A**) catalase (CAT), (**B**) superoxide dismutase (SOD), (**C**) glutathione peroxidase (GPx), and (**D**) glutathione reductase (GR) were assayed in clarified cell extracts obtained from cells incubated in the absence or presence of 1 mM PQ. The activities were determined by a coupled-enzyme assay as described in the Materials and Methods. A one-way ANOVA analysis was performed to detect significant differences between means using *p* < 0.05.

The results of the flow cytometry experiment with the FB2aox1-Gfp strain ruled out an increase in the expression of Aox1 under the oxidative stress induced by PQ. However, several reports have shown that the incubation of cells with PQ or H_2_O_2_ induces the expression of several proteins involved in the stress response [27,43,44]. Based on this information, the activities of four enzymes belonging to the *U. maydis* antioxidant system were assayed (Figure 4). In addition, a potential effect of Aox1 on the amount of enzyme was studied using three *U. maydis* strains (FB2, FB2aox1Δ, and FB2Potef:aox1-Gfp). The results indicate that the stress produced by PQ in the three strains did not affect, within the experimental error, the activities of the detoxifying enzymes catalase, SOD, GPx, and GR (Figure 4). An effect opposite to that expected was observed with catalase activity in the FB2aox1Δ and FB2Potef:aox1-Gfp mutants. In the presence of PQ, there was a decrease in the activity, although the difference was not statistically significant (Figure 4A). Taken together, these results strongly suggest that the stress induced by PQ in *U. maydis* cells did not modify the expression, at least at the protein level, of the main components of the antioxidant system (e.g., Aox1 and the other four enzymes).

### 3.6. Glutathione Content in Cells Incubated in the Presence of Paraquat

**Figure 5:**
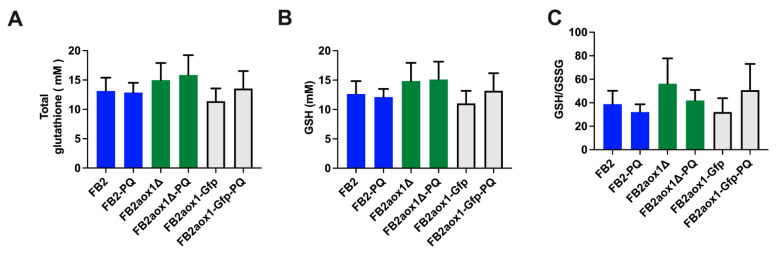
Determination of glutathione. Cells were incubated for 4 h in the presence or absence of 1 mM PQ. Then, the total glutathione (GSH + GSSG) was determined as described in the Materials and Methods. (**A**) Total glutathione, (**B**) GSH, (**C**) GSH/GSSG ratio. The bars represent the error standard of at least four independent experiments (*n* = 4). One-way ANOVA analysis was performed to detect significant differences between means using *p* < 0.05.

The third level of defense against ROS is the presence of organic compounds that remove radicals in reactions not catalyzed by enzymes. Among these substances, the most abundant in cells is glutathione. The concentration of this compound ranges between 0.4 and 20 mM [45]. In general, the GSH is much higher than the GSSG, so that the GSH/GSSG ratio is between 10 and 50 [34]. In the presence of oxidative stress, GSH is consumed and the GSH/GSSG ratio decreases. Since there were no changes in the activities of the Aox1 and antioxidant enzymes, it was important to follow the variations in the concentration of GSH, GSSG, and total GSH in cells incubated under different conditions (Figure 5). In addition, calculation of the GSH/GSSG ratio will provide information on the capacity of cells to contend with oxidative stress. Cells were incubated in the presence or absence of 1 mM PQ, centrifuged, and resuspended with 1 M HClO_4_. After 5 min incubation on ice, the suspension was centrifuged and the supernatant neutralized with 2 M KOH. The sample was centrifuged and an aliquot of the supernatant was used to determine GSSG, and another sample was used for total glutathione (GSH + 2GSSG). Figure 5 shows that the intracellular concentration of total glutathione was the same in cells incubated for 4 h in the presence or absence of PQ. Additionally, there was no difference in the glutathione content when the strains were compared (Figure 5A). Using the three strains for the calculation, we found that the concentration of GSH without or with PQ were 12.8 ± 1.6 mM and 13.5 ± 1.2 mM, respectively. As expected, the concentration of GSH was approximately 50 times higher than the concentration of GSSG either in the presence or the absence of PQ (52.2 ± 9.0 vs. 53.4 ± 8.6). For FB2 and FB2aox1Δ, there was a minor decrease in the GSH/GSSG ratio when cells were treated with PQ, although the difference was not statistically significant (Figure 5C). 

## 4. Discussion

Aerobic metabolism and exposure to harmful environmental conditions promote the production of ROS and the damage of DNA, proteins, and lipids, resulting in cell death [40,41,46]. As a safeguard against the increase in ROS levels, fungal and plant cells contain the alternative oxidase (Aox) [14,15,17,28,47]. Therefore, a working hypothesis is that cells containing the AOX should be more resistant to conditions that produce oxidative stress. In a previous study, we showed that this enzyme was not involved in the survival of *U. maydis* to high or low temperatures, the recovery of cells from desiccation, survival to salt stress, or the infection of the corn plant [15,17,48]. However, the exposure of *U. maydis* to oxidative stress has not been tested. The incubation of cells in the presence of PQ increased the production of ROS in both mitochondria and cytosol. A consequence of the higher production of H_2_O_2_ in the cytosol was the efflux of this substance into the extracellular space. Similar rates of H_2_O_2_ production were observed in the wild-type and mutant lacking Aox1. As expected, PQ negatively affected cell viability and the number of cells that can enter the replicative cycle and survive decreased to less than 5%. Because the extent of cell death was the same with or without the presence of the mitochondrial Aox1, the participation of this enzyme in the response of *U. maydis* to oxidative stress can be ruled out. Our results suggest that the increased levels of ROS induced extensive damage of DNA, producing apoptosis or necrosis when cells were forced to go through the cell cycle [23,26,27,44,49]. The FB2 and FB2aox1Gfp strains incubated in the presence of PQ maintained the same respiratory activity during the 4 h of incubation, indicating that the classic electron transport chain (complexes I, II, III, and IV) was not affected by the increase in ROS production. Regarding the Aox1, at the end of 4 h incubation, a small increase in the cyanide-resistant respiratory activity was observed. This relative increase in activity was the same in the strains, FB2 and FB2aox1Gfp. However, fluorescence microscopy and flow cytometry showed that the amount of Aox1-GFP in the cells was the same in the presence or absence of PQ, suggesting that the higher activity of Aox1 might be due to the activation of the enzyme. Our results contrast with the large amount of evidence indicating an increase in the expression of AOX during oxidative stress [28,50]. To explain the two results, we propose a connection between the inhibition of the respiratory chain by ROS and the increase in the AOX expression. The mitochondrial retrograde signaling might be responsible for the link between the nucleus and mitochondria. According to this hypothesis, if the stress affects the mitochondrial respiratory chain, a higher expression of AOX is expected. Since the respiratory activity was not affected in *U. maydis*, changes in Aox1 expression should be minimal. In support of this proposal, the incubation of *U. maydis* in the presence of antimycin A, an inhibitor of complex III, produced an increment in the amount of Aox1 [6]. 

It has been shown that under oxidative stress conditions, there is an increase in the activity of several enzymes including the GR and SOD [51] and changes in the concentration of GSH and GSSG. Within the experimental error, the activities over time were constant for all enzymes tested, GR, GPx, SOD, and catalase. Furthermore, the changes in the concentration of GSH and GSSG were minimal, so the GSH/GSSG ratio decreased very little. Taken together, the results indicate that the capacity of the antioxidant system in *U. maydis* including enzymes and metabolites is quite robust and can counteract some changes due to the oxidative stress. In spite of this capacity, the system is unable to prevent the damage produced to some molecules such as mitochondrial DNA, which results in cell death when they are forced to enter the replicative cycle [40]. The present study also showed that *U. maydis* Aox1 was not associated with the response of the cell against oxidative stress. In order to assign a function to this enzyme, more studies have to be conducted. 

## 5. Conclusions

PQ induces damage in *U. maydis* independent of the expression levels of Aox1. The activities of SOD, CAT, GPx, and GR did not show any significant change after incubation with PQ, while FB2aox1-Gfp cells showed mitochondrial fragmentation. Furthermore, incubation with PQ resulted in detention of the cell cycle, probably as a consequence of nuclear and mitochondrial DNA damage. Our results and previously published studies suggest that *U. maydis* Aox1 is not involved in the response to saline, thermic, pH, dehydration, or oxidative stresses. Therefore, it is necessary to perform more experiments at various stages of the cell cycle to assign a role to Aox1 in U. maydis. 

## Data Availability

Not applicable.
